# Equal access in urban spaces: Gendered perspectives and actions for greater equity

**DOI:** 10.1038/s44172-026-00753-x

**Published:** 2026-08-14

**Authors:** Natasha Watson

**Affiliations:** Buro Happold Ltd., Bath, UK

**Keywords:** Civil engineering, Science, technology and society

## Abstract

Natasha Watson, an engineering practitioner, synthesises how caregiving, safety, and male-centred design shape the navigation of and access to our urban environments for women and girls. Drawing on interdisciplinary literature, case studies, and existing gender mainstreaming frameworks, she explored what can be done to make cities more equitable for all.

## Teaser

In this article, I explore how urban design perpetuates gender inequity through neglecting the specific ways women experience the built environment. The women-specific dimensions are women’s disproportionate role as caregivers, their heightened perceptions of safety and exposure to harassment, and the persistence of a default-male approach in planning and design. These factors restrict women’s mobility, access to education and employment, and sense of belonging in public spaces. Drawing on interdisciplinary literature, case studies, and existing gender mainstreaming frameworks, I translate theory into practical actions for built environment professionals which covers participatory design processes, gender-sensitive planning interventions across transport and public space and increasing workforce diversity within the sector. Ultimately, I argue that gender equity as a systemic design responsibility and that professionals must actively apply existing tools and knowledge to create inclusive, safe, and accessible urban environments for all.

## Introduction

Our collective action as built environment professionals has been stymied by our lack of awareness of how our cities are designed in a way that negatively impacts women’s mobility, access to education, finances, sense of belonging, and safety. As a result, we are preventing ourselves from achieving Gender Equality, which is necessary for a peaceful, prosperous and sustainable future^1^.

Women occupy only 10% of the highest-ranking roles (technical and non-technical) in architecture and urban planning firms globally^2^, and only 13% of engineers in the UK are women^3^. Gender-awareness is a valid strategy to highlight the systemic inequity of women as a whole^4^, and the lack of appropriate gender representation within the built environment means that the industry is missing the wealth of knowledge that comes from a diverse lived experience through gender and its intersectionality with race, sexuality, class, and other socioeconomic characteristics. This lack of diverse experience hinders built environment professionals from addressing increasingly complex environmental and societal challenges.

I will consider this topic through the design of our urban environments. Cities have been historically planned and designed to reflect biases in our society, either through a lack of consideration for the different ways people use our cities, or through the perpetuation of prejudices or through a lack of representation of women and girls in planning and design decisions^2^. Although there are many different facets of inequity, I will focus on how urban design perpetuates gender inequity.

In this article, I introduce how women navigate the built environment differently: their role as caregivers, their perception of safety, and their marginalisation caused by designing primarily around men’s needs and characteristics. I will then discuss how these issues can be mitigated through involving citizens in creating public spaces, using existing gender mainstreaming interventions illustrated with examples from across the globe, and increasing the diversity of the built environment workforce.

I draw from academic literature from a range of disciplines, existing toolkits and guides, case studies, and existing initiatives within the built environment. The novelty lies in the translation of feminist urban theory into actions for built environment professions, and I argue that accountability for inclusion lies with these professionals as it is our design decisions that are producing unequal outcomes.

## How did this come to be?

Typical spatial planning and zoning create divides between sections of society in terms of access to the public realm. I will cover three areas that are predominantly relevant to urban design and how women navigate cities differently: their role as caregivers, their perception of safety, and the default-male.

## Women as Caregivers

The unpaid labour of caregivers and homemakers still typically falls on women and girls^5^. The Covid19 pandemic exacerbated this issue, as mothers were nearly three times as likely to report that they took on a majority or all of the extra care work that came from school closures and childcare facility closure caused by Covid19 lockdowns compared to fathers^6^.

Caregivers and those who work paid labour (henceforth described as ‘workers’) navigate the urban environment differently. Workers typically leave residential spaces to go to the commercial zones and return at the end of their workday. Caregivers typically ‘trip chain’ to complete various tasks, such as running errands, transport children, visit relatives and buy groceries, making many smaller journeys^7^. They are also much more likely to walk and take public transport^8^, which can be an additional time burden on women. This has impacts on the economic and educational emancipation of women.

This has impacts on the economic and educational emancipation of women, either through additional travel expenses^9^, caregiving activities taking precedence over school attendance^10^, or impacting women’s paid labour hours^11^.

## Women and perception of safety

Of the 190 economies studied by the World Bank in 2024^12^ only 39 have laws prohibiting sexual harassment in public spaces. In their 2016 review of international published research and media, the FIA Foundation^13^ found that harassment occurs frequently in public spaces as women travel to and from work and school; and especially at public transportation hubs and on public transport itself. In 2025, nine years later, similar themes are still being seen. In a study commissioned by the Department for Transport in the UK, Steen et al^14^. found through surveys that 54% of women public transport users aged 18 to 34 had experienced sexual harassment of unwanted behaviours which made them feel upset, distressed or threatened in the past 12 months.

In addition to harassment and violence, the perception of danger is enough for women, girls, and people perceived as feminine in urban spaces to change their behaviour and how they navigate through those spaces to feel comfortable^7,15^. Gender-disaggregated data in the form of perception-based data gathered through engaging with women and girls can reveal patterns that objective crime statistics alone miss^16^. Typically, evidence of vandalism, lack of activity, lack of lighting and fear of entrapment are the most common aspects that make a space feel unsafe^17–20^.

The behaviours and actions that women and girls perform to navigate spaces that feel unsafe is broadly known as ‘safety work’^21^ and can manifest in dressing and behaving differently, taking longer routes to avoid certain areas, avoiding public transport and taking a car or taxi, or avoiding visiting certain places alone^22–24^. This can greatly hinder mobility in a city, logistically and financially, thereby removing women and girls’ access to employment and education. Furthermore, when this safety work interferes with an individual’s wellbeing and quality of life, it can be considered dysfunctional worry^25^.

The intersectionality of gender and other personal characteristics such as race, age, and sexual orientation impacts women’s perception of safety too^2,19,20^ In certain cultures, and locations, there are also familial and community restrictions on the movement of women and girls born from a belief that this is to ‘protect’ them^26^.

## The default-male

I use the phrase default-male to mean that designing for men and boys is considered ‘standard’ and designing for women is considered ‘other’^27^. In this article, I use default-male to mean that seemingly ‘neutral’ spaces typically favour men and boys. For example, although seemingly open for equal use by all genders, research centring on interviews with girls and women have found that they typically avoid spaces where boys and men are present such as parks and playing fields^2,19,20,28–30^ and public toilets^2,19,20,31,32^.

Spaces for play within our cities typically include a large open space, either a grass-covered field or a MUGA (multi-use games area). Although these are technically open to all genders, some studies that collect gender-disaggregated data suggest that these larger open spaces are considered spaces for boys by girls^28^, with girls’ use of the space determined and regulated by the presence of boys. Studies also suggest that boys are more likely to play in parks than girls^29^. with one study finding that 88% of teenagers using park facilities were boys, and for MUGAs, this value was 92%^33^. As these spaces are popular, but not inclusive, they have been written into planning and council policies within the UK^19^ and perpetuate the gender bias. Only through the gathering of gender-disaggregated data can the neglect of providing spaces for teenage girls be seen^33^. Play and access to outdoor spaces are crucial for development and wellbeing^34,35^, and so the lack of equity in access impacts the development and mental health of girls.

Public toilets may have an equal number of stalls for women and men; however, due to physiological differences, women are still at a disadvantage. Women typically need to urinate more often^31^ and, due to their menstrual cycle, also typically need to visit a bathroom more often to maintain menstrual hygiene^36^.

Additionally, due to women typically taking on care responsibilities, if public toilets are not big enough or poorly laid out, women are less likely to use them at all^37^.

In areas where there is limited access to public toilets on the whole, girls either miss school or have their learning disrupted during menstruation^36^, perpetuating educational and economic inequities in women. Large distances from the home, security, and lighting are also reasons why women are at greater risk of assault when visiting public toilets, especially at night^32^.

## How do we solve it?

We need societal change to truly solve gender inequity; however, as built environment professionals, we can ensure that we are involving citizens in creating public spaces through participatory strategies, use existing gender mainstreaming interventions, and increase the diversity of the built environment workforce.

## Participatory Strategies

Women are a diverse population and cultural, location, and other differences may mean that certain urban design interventions are more appropriate than others. Inclusive participation strategies are found in a vast majority of handbooks and guidance on gender planning^38^, enabling the collection of the gender-disaggregated data that they need to ensure interventions are of equal benefit to all genders.

Examples of participatory strategies are walk audits^39^ with a diverse group sharing their experience of affected streets and locations, workshops sharing experiences in the project location; storytelling^18,40^; collaborative prioritisation of issues^30,32,41^, and participant-led design proposals^2,19,20,42^. These will require high-quality facilitation to ensure that participants are engaged and heard^2,42^.

Participatory strategies with women and girls enable lived experiences to emerge and for gender-disaggregated data to be collected^2,42^. These strategies go beyond engagement, as co-creation and co-design as a key part of the process to ensure that the concerns of the participants are appropriately addressed in the solutions. By engaging the impacted community directly, place-specific concerns over access, visibility, and safety are captured and addressed.

## Gender mainstreaming interventions

Gender mainstreaming is, defined as a plan to “*improve the quality of public policies, programmes and projects, ensuring a more efficient allocation of resources [that increase] wellbeing for both women and men and the creation of a more socially just and sustainable society*”^43^. Some of the examples of gender mainstreaming interventions outlined below impact urban zoning, transport planning, and design features for pedestrian ways, parks and public toilets.

### Urban Zoning

A focus on understanding how a caregiver uses the built environment will address the disproportionate burden on women for caregiving responsibilities, the associated mobility, and access to work and education. At the city scale, this can look like zoning allowances for caring and employment/education and minimising travel distances required for ‘work’ and ‘caregiving’ responsibilities^2^. These services could vary from creches to access to clean water and fuelwood and will vary between cities and neighbourhoods^2^. The focus on mixed-use development and minimising travel distances aligns with the 15-minute city concept, where “*basic urban amenities need to be provided in close proximity to urban centres, and such should not disadvantage people in respect of their socioeconomic status or age*.”^44^. Predominantly to address health and sustainability issues, urban sprawl and a reliance on the car, implementing a 15-minute city will address gender inequity^45^.

Zoning and provisions that allow women to engage in income-generating opportunities whilst still managing care-giving responsibilities is an example where caregivers are prioritised in urban design. An example from Kolkata^46^, saw women-led poorer households and other vulnerable groups being awarded ground flats during an environmental resettlement programme to give them the opportunity to set up small businesses from their new homes. Another example from Argentina^2^ involving the redesign of the central square of La Favorita, saw the inclusion of a raised vantage point next to a new playground which allowed for women and caregivers to watch their children whilst participating in a community market where the women could vend.

### Transport Options and Trip Chaining

Transport requirements vary significantly between cities, suburbs, rural areas, and across the globe, however transport forecasting arguably still focuses on discrete individual journeys that are aggregated by categorizing individuals (e.g. commuters) or locations (e.g. central business districts). This method allows for quick assessments of large-scale trends and impacts. However, this method can be biased towards focusing on commuter flows as they are typically the largest volume flow from suburbs to a city centre and back again^47^. The alternative activity-based model can either focus on real user-profiles, or more likely, synthetic user-profiles and national models^48^. Although an activity-based model captures many more mobility flows, it is likely that commuter trips will still dominate due to volumes.

To ensure that a proportionate amount of funding is applied to commuting and caregivers, appropriate gender-sensitive planning and policy development, such as the 9 Ps approach from Peters^49^ will maximise the chances that the updates to a transport network will better serve all genders. In 2012, Barcelona developed an orthogonal bus network^50^ that enabled quicker access to a greater area of the city, replacing the previous network that was based on the radial historical tram network, that connected the outskirts of the cities with the city centre^51^. The new bus network reduces the priority on typical commuter patterns of travel, ensuring that 95% of the Barcelona population has a stop less than 300 metres from their home^52^. This network also better allows for trip chaining between different areas of Barcelona. Furthermore, adopting fare structures that do not penalise taking a higher number of shorter journeys, such as the TfL ‘hopper fare’^53^ in London, UK, and unlimited travel in a set period such as Stockholm’s flat rate^54^ reduces the economic burden of trip chaining.

### Increasing safety to overcome the default male thinking

Increasing the perception of safety of a space is multi-faceted as the stated feelings and behaviour of individuals can be different^17^. ‘Nocturnas’, a publication by Col·lectiuPunt6^18^ on the life of women nightshift workers in Barcelona, provides a collection of pragmatic interventions that can be made to the streetscape to increase feelings of safety. These interventions were gathered using feminist participatory action research, through activities such as workshops, and involving twenty-four women nightshift workers as co-researchers^18^.Increasing visibility on a streetscape is a widely accepted intervention, such as minimising corners and ‘hidden areas’ on routes, keeping street furniture and other elements at less than 1 m high and ensuring regular cleaning vegetation trimming to maintain vitality^18^. Additionally, maintaining a steady level of activity over 24 hours and increasing ‘eyes on the street’^13,18^ through initiatives such as night markets can increase the perception of safety within urban areas. Effective street lighting was also captured as a way of increasing feelings of safety^18^. Bassam^55^ expands on what ‘effective’ lighting is; discussing how uniform, warm-toned, lighting of around 10-20 lux can remove dark-pockets due to high contrasts between lit and unlit spaces, glare, and potentially feeling ‘overexposed’ and ‘surveilled’; especially for those whose sense of belonging in public spaces is already low.

There are also various ways to minimise perceived and real safety concerns with transport options. These are also along the lines of a steady level of activity and clear lines of sight, which can be achieved through scheduling to minimise waiting times and introducing request stops at night and improvements to stops and stations such as ensuring good quality lighting and transparency of structure^18^. Campaigns to raise awareness of sexual harassment and information about appropriate services can also increase feelings of safety amongst women^18^.

In some instances, women-only carriages may be appropriate. More than 40% of women in Tokyo have been groped at least once in their lifetime, with over 80% of these incidences happening on trains^56^ In response, several lines in Japan have women-only cars for certain journeys^57^, however, women-only spaces can have unintended consequences. Crowdsourced data regarding the women only spaces from Rio de Janeiro^58^ found that women-only spaces on trains and stations can normalise harassment outside of those spaces, as women who choose not to use them are seen as provoking harassment, and onlookers assign responsibility for any harassment to the victim.

Finally, feelings of safety within public and community toilets can be increased through appropriate design^59^. To tackle safety and accessibility concerns with public and community toilets, guidance for women-friendly toilets from Wateraid^59^ can be followed, which includes practical steps for privacy, safety, accessibility, management and access for all users. These include design aspects such as no shared walls between toilet blocks for men and women, balancing privacy and safety in terms of visibility of entrances and conducting accessibility and safety audits with the users to capture nuanced specific issues.

### Design interventions for access and visibility

Alongside addressing safety concerns, specific interventions related to toilet provisions, parks, and streetscapes can also be made by designers to increase the access and visibility of women within public spaces. In terms of number of toilets, the UK’s RSPH recommends a ratio of 2:1^60^ toilets for men and women to account for menstruation, clothing and anatomical differences.

With regards to public parks, they can be designed to minimise domination by men and boys, such as with the redesign of Plaza Aliar in Mendoza, Argentina^2^. Through working with the local women, the project team ran activities that led to a shared understanding of the challenges and priorities for the new plaza; and six solutions were developed and presented back to the community to gather feedback and for voting^2^. The winning proposal was comprised of several smaller spaces, that included a multi-use structure for exercise classes that felt less exposed for the women’s comfort^2^. In their planning recommendations for gender-sensitive design of public parks, Vienna^30^ provides guidance for what activities matter to girls, boys, and caregivers for smaller children, and the Make Space for Girls campaign in the UK provides resources and design ideas, such as walking loops, conversation areas, and public toilets^30^.

In pedestrian areas, the addition of handrails and the replacement of stairs with ramps^30^ can increase the access to caregivers looking after children, people with disabilities, and older adults. The provision of wider streets to allow for wheelchairs/pushchairs, and the addition of appropriate places to sit and rest or to watch children also increases the access to public spaces for these groups^30^. To support breastfeeding, urban design could involve frequently spaced seating with high backs and high sides for partial screening^61^. Within public buildings, the provision of nursing rooms, baby-changing rooms accessible to all genders, and play areas in waiting rooms can improve the experience of carers of young children within the built environment as well^29^. Finally, challenging the historical underrepresentation of women in public spaces is essential. There is evidence that there are more equitable gender attitudes in areas with more streets that are named after women^62^. Naming streets and places is linked to power^63^, and in the case of naming streets after women, it is a strategy for local governments to demonstrate their commitment to gender equity^64^. There is also greater normalisation of public breastfeeding with associated signage and public art^61^.

## Diversity and Allyship within Built Environment Professionals

Gender mainstreaming was first established as a major global strategy for the promotion of gender equality in 1995 at the United Nations World Conference on Women in Beijing^65^. Despite being a strategy for close to 30 years, and there being gender mainstreaming policies in place in a number of cities, its implementation is not commonplace in policy^66^ or urban design^67^.

Studies across the years have cited different reasons for the lack of uptake of gender mainstreaming, from lack of training, to logistical issues such as the need for monitoring and evaluation, to resistance in many forms (e.g. denial of there being a problem, disavowal of responsibility, inaction, appeasement, co-option and repression)^68–73^.

We can increase the implementation of gender mainstreaming in urban design by increasing the diversity of our workforce and creating space for our experiences as citizens to be heard alongside our knowledge as built environment professionals. Greater gender balance in political, corporate and financial leadership positions manifests in diversity of thought, risk tolerance, experiences and collaboration styles^74^; the qualities required to understand the breadth of needs and characteristics that need to be catered for in urban design.

From a professional perspective, as I stated at the beginning of this article, women’s representation within built environment professions is much lower than 50% on average globally. Over the last ten years, women have steadily only made up 35% of STEM graduates^75^; and professional women leave the built environment workforce either due to long working hours that impact work-family balance, and that changing working practices to suit work-family balance, such as access to flexible working; whether that’s phased returns, working around childcare, or tackling presenteeism and long hours^76,77^.

Women, and especially women of colour, who work in planning research have their lived experiences and knowledge undermined in the name of objectivity and neutrality, which harms the wellbeing of researchers and marginalises them^78^. This leads to their knowledge as citizens not being valued as they research urban planning.

Our industry needs to value social, environmental and care-focused knowledge to the same level as spatial decision-making. Readers of all genders can work towards redefining legitimacy and expertise in urban policy by having the courage to use readily available gender mainstreaming toolkits. These include the World Bank’s Handbook for Gender Inclusive Urban Planning Design^2^, Vienna’s ‘Gender Mainstreaming Made Easy’^30^, European Institute for Gender Equality’s resources on Gender Mainstreaming, the IMF’s Strategy towards mainstreaming gender^55,61^, the UN’s ‘Her City’ guidance^41^,and ‘Cities Alive Designing cities that work for women’ by ARUP, UNDP, and University of Liverpool^79^.

## Conclusions

Throughout this paper, I demonstrate that gender inequity is rarely the result of isolated design decisions, and instead the interaction between planning systems, professional norms, data limitations, and assumptions that shape how our urban environment is designed and managed. I also present potential interventions that can be implemented by built environment professionals, showing that they have the power to design with greater equity.

I do not identify new forms of gender exclusion but instead synthesise existing evidence and provide guidance for built-environment professionals to improve access to our urban spaces along gender lines. By connecting caregiving, perceived safety, and the ‘default male’ across citizen-led design, zoning, transport planning, public-space design, and professional culture, I reframe gender equity as a systemic design challenge rather than an issue for policy-makers only. By considering gender in everyday planning and design processes, rather than treating them as specialists or supplementary concerns, build environment professionals can better design safer, more accessible, and more inclusive cities.

Furthermore, I argue that a lack of progress is not a case of gender mainstreaming tools not being effective; it is a case of them not being used effectively by built environment professionals. I discuss that this ineffective use is either through a lack of awareness, a lack of gender-disaggregated and/or perception-based data, and a lack of workforce diversity.

Through reading articles such as this one and opening up the conversation, I believe that the development of gender-equitable urban spaces is more likely to be commonplace in the future.

## Supplementary information

Transparent Peer Review file

## Data Availability

No additional data were produced for this article

## References

[1] United Nations (n.d.) Gender Equality. Available at: https://www.un.org/sustainabledevelopment/gender-equality/ [Accessed: 8 April 2025].

[2] World Bank (n.d.) Handbook for Gender-Inclusive Urban Planning and Design. Available at: https://www.worldbank.org/en/topic/urbandevelopment/publication/handbook-for-gender-inclusive-urban-planning-and-design [Accessed: 8 April 2025].

[3] WISE Campaign (2022) Updated Workforce Stats September 2022. Available at: https://www.wisecampaign.org.uk/updated-workforce-stats-september-2022/ [Accessed: 8 April 2025].

[4] Harvard Business Review (2018) Women Benefit When They Downplay Gender. Available at: https://hbr.org/2018/07/women-benefit-when-they-downplay-gender [Accessed: 8 April 2025].

[5] Grosser, K. (2009) Corporate social responsibility and gender equality: Women as stakeholders and the European Union sustainability strategy. Business Ethics: A European Review, 18, pp.290-307. Available at: 10.1111/j.1467-8608.2009.01564.x [Accessed: 8 April 2025].

[6] OECD (2021) Caregiving in Crisis: Gender inequality in paid and unpaid work during COVID-19. OECD Policy Responses to Coronavirus (COVID-19), OECD Publishing, Paris, Available at: https://www.oecd.org/en/publications/2021/12/caregiving-in-crisis-gender-inequality-in-paid-and-unpaid-work-during-covid-19_85044948.html [Accessed: 8 April 2025].

[7] Vanderschuren, M. J., Phayane, S. R. & Gwynne-Evans, A. J. Perceptions of gender, mobility, and personal safety: South Africa moving forward. *Transportation Res. Rec.* **2673**, 616–627 (2019).

[8] Goel, R. et al. Gender differences in active travel in major cities across the world. *Transportation* **50**, 733–749 (2023). 37035250 10.1007/s11116-021-10259-4 PMC7614415

[9] NYU Wagner (2018) Pink Tax Report. Available at: https://wagner.nyu.edu/files/faculty/publications/Pink%20Tax%20Report%2011_13_18.pdf [Accessed: 8 April 2025].

[10] Nauges, C. & Strand, J. Water Hauling and Girls’ School Attendance: Some New Evidence from Ghana. *Environ. Resour. Econ.* **66**, 65–88 (2017).

[11] Office for National Statistics (2021) Unpaid Care by Age, Sex, and Deprivation, England and Wales. Available at: https://www.ons.gov.uk/peoplepopulationandcommunity/healthandsocialcare/socialcare/articles/unpaidcarebyagesexanddeprivationenglandandwales/census2021 [Accessed: 8 April 2025].

[12] World Bank Group (2024) Women, Business and the Law 2024. Open Knowledge Repository, Available at: https://openknowledge.worldbank.org/bitstreams/9bc44005-2490-41f8-b975-af35cbae8b9a/download [Accessed: 8 April 2025].

[13] FIA Foundation (2016) Safe and sound: International research on women’s personal safety on public transport. FIA Foundation Research Series, Paper 6. March. London: FIA Foundation. Available at: https://intalinc.org/wp-content/uploads/2021/03/safe-and-sound-report.pdf [Accessed: 29 April 2026].

[14] Steen, B., Graham, B., Link, S., Chabdu, A. & Jones, E. (2025) Personal safety on public transport. London: Department for Transport. Available at: https://assets.publishing.service.gov.uk/media/697338e6d8adeaf266040588/personal-safety-on-transport.pdf [Accessed: 29 April 2026].

[15] Boomsma, C. & Steg, L. Feeling safe in the dark: Examining the effect of entrapment, lighting levels, and gender on feelings of safety and lighting policy acceptability. *Environ. Behav.* **46**, 193–212 (2014).

[16] Beebeejaun, Y. Gender, urban space, and the right to everyday life. *J. Urban Aff.* **39**, 323–334 (2017).

[17] Ortiz Escalante, S. & Gutiérrez Valdivia, B. Planning from below: using feminist participatory methods to increase women’s participation in urban planning. *Gend. Dev.* **23**, 113–126 (2015).

[18] Col.lectiuPunt6, (2017) Nocturnas. The everyday life of women nightshift workers in the Barcelona Metropolitan Area. Available at: https://www.punt6.org/wp-content/uploads/2024/10/nocturnas_eng.pdf [Accessed: 16 April 2025].

[19] Walker, S. & Clark, I. (2023) Make Space for Girls: The research background (research draft). January. London: Make Space for Girls. Available at: https://cdn.prod.website-files.com/6398afa2ae5518732f04f791/63f60a5a2a28c570b35ce1b5_Make%20Space%20for%20Girls%20-%20Research%20Draft.pdf [Accessed: 29 April 2026].

[20] Yorkshire Sport Foundation (2022) Make Space for Us: Insight report. July. Available at: https://womeninsport.org/wp-content/uploads/2023/05/Make-Space-for-Us-Yorkshire-Sport-Foundation-2022.pdf [Accessed: 29 April 2026].

[21] Vera-Gray, F. & Kelly, L., (2020). Contested gendered space: Public sexual harassment and women’s safety work. In Crime and fear in public places (pp. 217-231). Routledge. Available at: 10.1080/01924036.2020.1732435 [Accessed: 26 April 2026].

[22] Petherick, N. Environmental design and fear: The prospect-refuge model and the university college of the Cariboo campus. *West. Geogr.* **10**, 89–112 (2026).

[23] García-Carpintero, M. Á, de Diego-Cordero, R., Pavón-Benítez, L. & Tarriño-Concejero, L. Fear of walking home alone’: Urban spaces of fear in youth nightlife. *Eur. J. Women’s. Stud.* **29**, 39–53 (2020).

[24] Ceccato, V. & Loukaitou-Sideris, A. Fear of Sexual Harassment and Its Impact on Safety Perceptions in Transit Environments: A Global Perspective. *Violence Women* **28**, 26–48 (2021). 10.1177/1077801221992874 PMC8564253 33656953

[25] Jackson, J. & Gray, E. Functional fear and public insecurities about crime. * Br. J. Criminol.* **50**, 1–22 (2010).

[26] Shah, S., Viswanath, K., Vyas, S. & Gadepalli, S., (2017). Women and transport in Indian cities. New Delhi, India: ITDP India, pp.10-11. Available at: https://safetipin.com/wp-content/uploads/2020/01/women-and-transport-in-indian-cities-safetipin-2017.pdf [Accessed 16 April 2025].

[27] Beauvoir, S. de. The Second Sex (eds Borde, C. & Malovany-Chevallier, S. Trans.). (Vintage Books, Original work published 1949) (2011).

[28] Rönnlund, M., (2015). Schoolyard stories: Processes of gender identity in a ‘children’s place’. Childhood, 22, pp.85-100. Available at: https://www.researchgate.net/publication/272361808_Schoolyard_stories_Processes_of_gender_identity_in_a_‘children’s_place’ [Accessed: 16 April 2025].

[29] Cohen, D. A., Williamson, S. & Han, B. Gender differences in physical activity associated with urban neighborhood parks: findings from the national study of neighborhood parks. *Women’s. health issues* **31**, 236–244 (2021). 33358644 10.1016/j.whi.2020.11.007 PMC8154653

[30] City of Vienna, (2011). Gender Mainstreaming Made Easy: A Manual. Available at: https://www.wien.gv.at/english/administration/gendermainstreaming/principles/manual.html [Accessed 16 April 2025].

[31] Mueller, E. et al. Gender differences in 24-hour urinary diaries of asymptomatic North American adults. * J. Urol.* **173**, 490–492 (2005). 15643226 10.1097/01.ju.0000149947.28100.cd

[32] UNICEF, (2018). Female-friendly public and community toilets: a guide for planners and decision makers. Available at: https://apo.org.au/node/247186 [Accessed 16 April 2025].

[33] Make Space for Girls (2023) “Most parks have more facilities for dog waste than for teenage girls”: Parkwatch report. September. London: Make Space for Girls. Available at: https://cdn.prod.website-files.com/63989e164f27a3461de801f1/650959220c2ba45328a16390_MSFG_PW_Report_FINAL.pdf [Accessed: 29 April 2026].

[34] Vygotsky, L. S., & Cole, M. (1978). Mind in society: The development of higher psychological. Cambridge, MA: Harvard University.

[35] Abraham, A., Sommerhalder, K. & Abel, T. Landscape and well-being: A scoping study on the health-promoting impact of outdoor environments. *Int. J. Public Health* **55**, 59–69 (2010). 19768384 10.1007/s00038-009-0069-z

[36] Greed, C., (2017). Taking women’s ‘different’ bodily functions into account, particularly menstruation in sanitation provision. Available at: https://repository.lboro.ac.uk/articles/Taking_women_s_different_bodily_functions_into_account_particularly_menstruation_in_sanitation_provision/9589199/files/17228285.pdf [Accessed 27 April 2026].

[37] Lewkowitz, S. & Gilliland, J. A feminist critical analysis of public toilets and gender: A systematic review. *Urban Aff. Rev.* **61**, 282–309 (2025).

[38] Tummers, L. & Wankiewicz, H., (2020). Gender mainstreaming planning cultures: Why ‘engendering planning’ needs critical feminist theory. GENDER–Zeitschrift für Geschlecht, Kultur und Gesellschaft, 12, pp.7-8. Available at: 10.3224/gender.v12i1.02 [Accessed 16 April 2025].

[39] Safe Routes to School National Partnership, (2018). Let’s Go For A Walk: A Toolkit for Planning and Conducting a Walk Audit. [online] Available at: https://www.communitycommons.org/entities/fe533457-ce51-4279-849f-2c354a79cd23 [Accessed 16 April 2025].

[40] Machielse, W., (2015). Perceived safety in public spaces: A quantitative investigation of the spatial and social influences on safety perception among young adults in Stockholm. Available at: https://www.diva-portal.org/smash/get/diva2:826168/FULLTEXT01.pdf [Accessed 16 April 2025].

[41] UN-Habitat, (2021). Her City: A Guide for Cities to Sustainable and Inclusive Urban Planning and Design Together with Girls. [online] Available at: https://unhabitat.org/her-city-a-guide-for-cities-to-sustainable-and-inclusive-urban-planning-and-design-together-with [Accessed 16 April 2025].

[42] Col·lectiu Punt 6 (2021). Brief guide to design a participatory process from a gender perspective. Barcelona: MedCities, COHESIMED II Project. Available at: https://medcities.org/wp-content/uploads/2022/01/BriefGuide_ParticipationGender_final.pdf [Accessed: 29 April 2026].

[43] Council of Europe, (2025). What is gender mainstreaming? Available at: https://www.coe.int/en/web/genderequality/what-is-gender-mainstreaming [Accessed 16 April 2025].

[44] Moreno, C., Allam, Z., Chabaud, D., Gall, C. & Pratlong, F. Introducing the “15-Minute City”: Sustainability, resilience and place identity in future post-pandemic cities. *Smart cities* **4**, 93–111 (2021).

[45] Willberg, E., Fink, C. & Toivonen, T. The 15-minute city for all?–Measuring individual and temporal variations in walking accessibility. *J. Transp. Geogr.* **106**, 103521 (2023).

[46] Asian Development Bank, (2011). Gender Mainstreaming Case Studies: India. Available at: https://www.adb.org/sites/default/files/publication/29934/gender-mainstreaming-case-studies-india.pdf [Accessed 16 April 2025].

[47] Banister, D. The sustainable mobility paradigm. *Transp. policy* **15**, 73–80 (2008).

[48] Department for Transport, (2023). Transport user personas: understanding different users and their needs. Available at: https://www.gov.uk/guidance/transport-user-personas-understanding-different-users-and-their-needs [Accessed 16 April 2025].

[49] Esariti, L., Putri, K. & Wahyono, H. Application of importance performance analysis (IPA) method for gender based public transportation planning in corridor I bus rapid transit Semarang city. *J. Pengemb. Kota* **8**, 58–66 (2020).

[50] Transports Metropolitans de Barcelona, (2019). What it is and the completed bus network. Available at: https://www.tmb.cat/en/about-tmb/transport-network-improvements/new-bus-network/evolution [Accessed 16 April 2025].

[51] Ajuntament de Barcelona, (2025). Xarxa de tramvies de Barcelona l’any 1888. Available at: https://bcnmobilitat.wordpress.com/tag/tram/ [Accessed 16 April 2025].

[52] Transports Metropolitans de Barcelona, (2019a). New bus network. Available at: https://www.tmb.cat/en/get-to-know-tmb/transport-network-improvements/new-bus-network [Accessed 16 April 2025].

[53] London City Hall, (2016). The Mayor’s one hour ‘Hopper’ fare. Available at: https://www.london.gov.uk/programmes-strategies/transport/mayors-one-hour-hopper-fare [Accessed 16 April 2025].

[54] Stockholm Public Transport (SL) (n.d.) Single journey tickets. Available at: https://sl.se/en/fares-and-tickets/visitor-tickets/single-journey-tickets [Accessed: 29 April 2026].

[55] Bassam, N., (2025) Not All Light Is Safe. Available at: https://genderedcity.org/feminist-urban-blueprint/f/not-all-light-is-safe. [Accessed: 22 April 2026].

[56] Tokyo Metropolitan Government, (2023). Press release on measures against groping. Available at: https://www.metro.tokyo.lg.jp/tosei/hodohappyo/press/2023/12/25/09.html [Accessed 16 April 2025].

[57] Osaka Metro, (2022). Information on women-only cars. Available at: https://subway.osakametro.co.jp/en/guide/usage/female_vehicle/220401woman_syaryou.php [Accessed 16 April 2025].

[58] World Bank, (2020). Demand for “Safe Space”: Avoiding Harassment and Complying with Norms. Available at: https://openknowledge.worldbank.org/entities/publication/b61813eb-9124-5f10-b722-9bfad188c8b4 [Accessed 16 April 2025].

[59] WaterAid, (2018) Female-Friendly Public and Community Toilets: a guide for planners and decision makers. Available at: https://www.wateraid.org/washmatters/resources/female-friendly-public-and-community-toilets [Accessed: 8 April 2025].

[60] Royal Society for Public Health, (2019_ Taking the P***. The decline of the Great British toilet. Available at: https://www.rsph.org.uk/media/filer_public/0a/f7/0af7df7c-2fd8-4ca7-a81a-1e4fecd226d1/taking_the_p.pdf [Accessed: 8 April 2025].

[61] Interior Health (n.d.) Breastfeeding Friendly Public Spaces Toolkit. Available at: https://www.interiorhealth.ca/sites/default/files/PDFS/breastfeeding-friendly-public-spaces-toolkit.pdf (Accessed: 8 April 2025).

[62] Gutiérrez-Mora, D. & Oto-Peralías, D. Gendered cities: Studying urban gender bias through street names. *Environ. Plan. B: Urban Analytics City Sci.* **49**, 1792–1809 (2022).

[63] Bancilhon, M., Constantinides, M., Bogucka, E. P., Aiello, L. M. & Quercia, D. Streetonomics: Quantifying culture using street names. *PLoS ONE* **16**, e0252869 (2021). 34191817 10.1371/journal.pone.0252869 PMC8244867

[64] Ajuntament de Barcelona, (2024). New Streets Named After Women. Available at: https://www.barcelona.cat/infobarcelona/en/tema/democratic-memory/new-streets-named-after-women_1383588.html [Accessed: 8 April 2025].

[65] United Nations, (2002). Gender Mainstreaming: An Overview. [pdf] Available at: https://www.un.org/womenwatch/osagi/pdf/e65237.pdf [Accessed 16 Apr. 2025].

[66] Engeli, I. & Mazur, A. Taking implementation seriously in assessing success: The politics of gender equality policy. *Eur. J. Politics Gend.* **1**, 111–129 (2018).

[67] Horelli, L. Engendering urban planning in different contexts–successes, constraints and consequences. *Eur. Plan. Stud.* **25**, 1779–1796 (2017).

[68] Moser, C. & Moser, A. Gender mainstreaming since Beijing: a review of success and limitations in international institutions. *Gend. Dev.* **13**, 11–22 (2005).

[69] Lombardo, E. & Mergaert, L. Gender mainstreaming and resistance to gender training: A framework for studying implementation. *NORA-Nord. J. Feminist Gend. Res.* **21**, 296–311 (2013).

[70] Rausch, M., Debroux, T. & de Craene, V., (2020). Feminist claims vs. administrative reality: Situating gender mainstreaming in urban planning within the academic discourse–A case study of Vienna, Austria (Niet gepubliceerde masterscriptie). Vrije Universiteit Brussel, Brussel, pp.2020-11.Available at: https://urbanstudies.brussels/sites/default/files/2020-11/2020_Rausch.pdf [Accessed 16 April 2025].

[71] Vida, B. Policy framing and resistance: Gender mainstreaming in Horizon 2020. *Eur. J. Women’s. Stud.* **28**, 26–41 (2021).

[72] Elomäki, A. & Ylöstalo, H. Gender budgeting in the crossroads of gender policy and public financial management: The Finnish case. *Public Money Manag.* **41**, 516–526 (2021).

[73] Flood, M., Dragiewicz, M. & Pease, B. Resistance and backlash to gender equality. *Aust. J. Soc. Issues* **56**, 393–408 (2021).

[74] Goyal, R. & Sahay, R., (2023). Integrating Gender into the IMF’s Work. International Monetary Fund. Available at: https://www.imf.org/-/media/Files/Publications/GNS/2023/English/GNSEA2023001.ashx [Accessed 16 April 2025].

[75] UNESCO (n.d.) Girls’ and women’s education in science, technology, engineering and mathematics (STEM). Available at: https://www.unesco.org/en/gender-equality/education/stem [Accessed: 29 April 2026].

[76] Baker, M., Ali, M. & Crawford, L. What do women want? Workplace attraction and retention factors for women in construction. *Int. J. Constr. Manag.* **24**, 270–280 (2024).

[77] Senaratne, S., Jayakodi, S., Pascoe, R. D. & Atkins, A. Challenges and strategies for the retention of female construction professionals. *Buildings* **15**, 2187 (2025).

[78] Beebeejaun, Y. Race, gender, and positionality in urban planning research. *Int. J. Qualitative Methods* **21**, 16094069211066167 (2022).

[79] Arup, University of Liverpool, and United Nations Development Programme (2021) Cities Alive: Designing cities that work for women. Available at: https://www.arup.com/insights/cities-alive-designing-cities-that-work-for-women.

