# Supplementary material for: Equal access in urban spaces: Gendered perspectives and actions for greater equity
Source: Commun Eng. 2026 Aug 14;5:149. doi: 10.1038/s44172-026-00753-x (PMC13476247; doi:10.1038/s44172-026-00753-x)
Supplement: Supplementary file 1 [file 44172_2026_753_MOESM1_ESM.pdf]

nature portfolio

Peer Review File

Equal Access in Urban Spaces: Gendered Perspectives and Actions for Greater Equity

Corresponding Author: Dr Natasha Watson

This file contains all reviewer reports in order by version, followed by all author rebuttals in order by version.

Version 0:

Reviewer comments:

Reviewer #1

(Remarks to the Author)
This paper aims to explore how women navigate areas differently, focussing on how urban design can perpetuate gender inequality for women in their roles as caregivers, in their perceptions of safety, and in the default of the male in urban design. It then aims to provide recommendations for interventions to improve gender equity. From what I can gather, the key points are that 1. There needs to be an increase in workforce diversity, 2. The involvements of citizens in creating spaces, and 3. Use gender mainstreaming toolkits that already exist.

This paper is likely to be of interest to others in the field, but it currently lacks significance in its current form. The recommendations that the paper aims to suggest are lost, which could be due to the structure of the paper, and its overall underdevelopment throughout. This paper has potential, but currently lacks the depth, detail, flow, and significance for publication.

A concern throughout is the number of unsupported claims that require citations. For example:
• At lines 62-64
• Lines 76-77
• Lines 96-98
• Lines 115-117
• Lines 130-133
• Lines 158-161
• Lines 181-183
Where these lines consist of suggestions or recommendations, there must be an evidence-base for that, hence citations.

Each of the sections currently feel underdeveloped. For example, the women and perception of safety is only five sentences, and nine lines long. Ideas could be developed a little further, with the inclusion of more theory. Women's safety and hidden safety work (https://www.tandfonline.com/doi/full/10.1080/01924036.2020.1732435), and dysfunctional worry (https://www.jstor.org/stable/43612845) when these hidden safety work behaviours become detrimental to women's quality of life and wellbeing. Also consider "what makes a park feel safe or unsafe" (https://eprints.whiterose.ac.uk/id/eprint/194214/1/Parks Report FINAL 7.12.2022.pdf).

The inclusion of statistics at appropriate places would also help with highlighting the starkness of the inequalities. See for example Parkwatch by Make Space for Girls (https://cdn.prod.website-files.com/63989e164f27a3461de801f1/650959220c2ba45328a16390_MSFG_PW_Report_FINAL.pdf), 94% of park facilities in England are skate parks, MUGAs, BMX tracks, of which boys are 90% of the users. And the comments here written into council policies is also of interest!

In thinking about planning for caregivers, proximity to work and services appears important, so how does the paper engage with the concept of the 15 minute city/neighbourhood?

Developing the participatory strategies section would also be beneficial. Dig into co-design and co-creation of spaces with citizens and find previous examples of this put into action in urban planning, use some case studies to give your paper some strong groundings.

I like that suggestions are often followed with examples (e.g Kolkata), which is helpful. However, consider integrating these more cohesively into the main points and expanding upon them to strengthen the points and recommendations.

I also like that you give a good round up of why these issues are yet to be solved, from line 221, which then leads nicely into what we can do, but a stronger conclusion is needed here. Make clear what the recommendations are, reiterate them in your conclusion, and why. Explicitly state what is novel about your paper, what it brings to the field, as this is currently not clear and lost in the paper.

Overall, I think that the paper has great potential, but that it is currently underdeveloped and somewhat disjointed. Each section would benefit from deeper development and a stronger evidence base to support your arguments more effectively. It would also benefit from an overarching comment about methodology, that speaks to the rigour or exploratory nature of the review/selection of interventions included. The ideas would benefit from more depth, and a stronger theoretical foundation. Bring in relevant theories throughout and ensure that all claims, no matter how small, are supported by references.

Dr Jennie Gray and Dr Anna Barker, University of Leeds

Open Access This Peer Review File is licensed under a Creative Commons Attribution 4.0 International License, which permits use, sharing, adaptation, distribution and reproduction in any medium or format, as long as you give appropriate credit to the original author(s) and the source, provide a link to the Creative Commons license, and indicate if changes were made.
In cases where reviewers are anonymous, credit should be given to 'Anonymous Referee' and the source.
The images or other third party material in this Peer Review File are included in the article's Creative Commons license, unless indicated otherwise in a credit line to the material. If material is not included in the article's Creative Commons license and your intended use is not permitted by statutory regulation or exceeds the permitted use, you will need to obtain permission directly from the copyright holder.
To view a copy of this license, visit https://creativecommons.org/licenses/by/4.0/

Response to Reviewers

Changes within this Review

260720 - For ‘Equal Access in Urban Spaces: Gendered Perspectives and Actions for Greater Equity’

Reviewer Comments	Author Responses
1. You don't need to repeat the points made in the conclusion, this doesn't add value to the piece. I'd recommend deleting your list repeating the points made earlier, and focussing attention in the Conclusion section on your final points.	List removed. Please see revised manuscript for highlighted changes.
2. I'd recommend changing your language from "This paper does not identify new forms of gender exclusion..." "This paper argues..." to "I do not identify.." and "I argue...". And check throughout the piece for other example and replace where necessary (Another example is in the abstract). It's more powerful to write in the first person.	Language adjusted to be first person statements. Please see revised manuscript for highlighted changes.
3. The final sentence in the Conclusion is repeated. Please delete.	Repeated sentence deleted. Please see revised manuscript for highlighted changes.
4. Note there are quite a number of typos. Many will be removed in typesetting, but I'd recommend using spellchecker and going through the text yourself for reassurance.	Spellchecker within Microsoft Word used. These typos were not captured within the highlighted changes.
5. Nature portfolio is committed to supporting clear and unbiased communication of research, consistent with our commitment to embedding inclusive principles in publishing practices. Please ensure that your manuscript follows the principles set out in our inclusive language guide (https://resource-cms.springernature.com/springer-cms/rest/v1/content/26176720/data/v2)	There were several uses of the words ‘female’ and ‘male’ which was informed by the classifications from the literature being referenced. Although in some instances, the discussion was about physiological differences; these are still not wholly about biological sex, and so in all instances, ‘female’ was replaced with ‘women’ and ‘male’ replaced with ‘men’ to keep the paper as an exploration of gender.<br><br>People-first descriptions replaced other terms; such as ‘older people’ replacing the term ‘the elderly’; ‘women-led poorer households’ replacing ‘poorer women-led households’, and using more specific terms such as ‘people with disabilities’ rather than ‘those with disabilities’.<br><br>Finally, I replaced ‘normalising the presence of women’ with ‘challenging the historical underrepresentation’ to ensure that the phrasing didn’t suggest that women in the urban environment was abnormal.<br><br>Please see revised manuscript for highlighted changes.

Previously addressed changes (amendments in red)

250527 - For 'Equal Access in Urban Spaces: Gendered Perspectives and Actions for Greater Equity'

Thanks go to Dr Jennie Gray and Dr Anna Barker, University of Leeds, for their review of this article, '*Equal Access in Urban Spaces: Gendered Perspectives and Actions for Greater Equity*'.

Responses to their comments are below.

Reviewer Comments	Author Responses
This paper aims to explore how women navigate areas differently, focussing on how urban design can perpetuate gender inequality for women in their roles as caregivers, in their perceptions of safety, and in the default of the male in urban design. It then aims to provide recommendations for interventions to improve gender equity. From what I can gather, the key points are that 1. There needs to be an increase in workforce diversity, 2. The involvements of citizens in creating spaces, and 3. Use gender mainstreaming toolkits that already exist.	No comments
This paper is likely to be of interest to others in the field, but it currently lacks significance in its current form. The recommendations that the paper aims to suggest are lost, which could be due to the structure of the paper, and its overall underdevelopment throughout. This paper has potential, but currently lacks the depth, detail, flow, and significance for publication.	Paper restructured
A concern throughout is the number of unsupported claims that require citations. For example:<br>• At lines 62-64<br>• Lines 76-77<br>• Lines 96-98<br>• Lines 115-117<br>• Lines 130-133<br>• Lines 158-161<br>• Lines 181-183<br>Where these lines consist of suggestions or recommendations, there must be an evidence-base for that, hence citations.	These claims were as follows; with the appropriate citation.<br><br>• At lines 62-64 – default male spaces. This section has been re-written and supported by sources<br>• Lines 76-77 – Public toilet sizes. This section has been re-written and supported by sources<br>• Lines 96-98 – Zoning allowances and minimising travel distances. This section has been re-written and supported by sources.<br>• Lines 115-117 – Small scale street interventions. This section has been re-written and supported by sources.<br>• Lines 130-133 – Overall introduction to transport options and trip chaining. This section has been re-written and supported by sources.<br>• Lines 158-161 – Minimise safety concerns with transport options. This section has been re-written and supported

	by sources.<br>• Lines 181-183 – Steady level of activity on a street scape<br><br>Due to the structuring of the paper, some of these sentences have now been amended
Each of the sections currently feel underdeveloped. For example, the women and perception of safety is only five sentences, and nine lines long. Ideas could be developed a little further, with the inclusion of more theory. Women's safety and hidden safety work (https://www.tandfonline.com/doi/full/10.1080/01924036.2020.1732435), and dysfunctional worry (https://www.jstor.org/stable/43612845) when these hidden safety work behaviours become detrimental to women's quality of life and wellbeing. Also consider "what makes a park feel safe or unsafe" (https://eprints.whiterose.ac.uk/id/eprint/194214/1/Parks Report FINAL 7.12.2022.pdf).	This paragraph has been developed further using the sources suggested and building on this topic.
The inclusion of statistics at appropriate places would also help with highlighting the starkness of the inequalities. See for example Parkwatch by Make Space for Girls (https://cdn.prod.website-files.com/63989e164f27a3461de801f1/650959220c2ba45328a16390_MSFG_PW_Report_FINAL.pdf), 94% of park facilities in England are skate parks, MUGAs, BMX tracks, of which boys are 90% of the users. And the comments here written into council policies is also of interest!	This paragraph has been developed further using the sources suggested and building on this topic.
In thinking about planning for caregivers, proximity to work and services appears important, so how does the paper engage with the concept of the 15 minute city/neighbourhood?	15 minute cities has been addressed within the paper
Developing the participatory strategies section would also be beneficial. Dig into co-design and co-creation of spaces with citizens and find previous examples of this put into action in urban planning, use some case studies to give your paper some strong groundings.	The paper has been restructured for this section to be better elaborated; and the participatory methods for Plaza Aliar and for the creation of the Nocturnas publication have been stated.
I like that suggestions are often followed with examples (e.g Kolkata), which is helpful. However, consider integrating these more cohesively into the main points and expanding upon them to strengthen the points and recommendations.	This has been accounted for within the restructuring
I also like that you give a good round up of why these issues are yet to be solved, from line 221, which then leads nicely into what we can do, but a stronger conclusion is needed here. Make clear what the recommendations are, reiterate them in your conclusion, and why. Explicitly state what is novel about your paper,	Bulletpoint actions have been added within the conclusion.<br><br>Amendment: This bulletpoint list was removed

what it brings to the field, as this is currently not clear and lost in the paper.	
Overall, I think that the paper has great potential, but that it is currently underdeveloped and somewhat disjointed. Each section would benefit from deeper development and a stronger evidence base to support your arguments more effectively. It would also benefit from an overarching comment about methodology, that speaks to the rigour or exploratory nature of the review/selection of interventions included. The ideas would benefit from more depth, and a stronger theoretical foundation. Bring in relevant theories throughout and ensure that all claims, no matter how small, are supported by references.	This author has restructured and added more commentary about how this article is an exploration and synthesis of literature from a range of disciplines into actions for built environmental professionals.
